# Mel-CAM Expression in Common Oral Carcinomas

**DOI:** 10.30476/DENTJODS.2019.77873.0

**Published:** 2020-09

**Authors:** Nafise Shamloo, Nasim Taghavi, Samira Behrad, Ali Dehghani Nazhvani

**Affiliations:** 1 Dept. of Oral and Maxillofacial Pathology, School of Dentistry, Shahid Beheshti University of Medical Sciences, Tehran, Iran; 2 Dept. of Oral and Maxillofacial Pathology, School of Dentistry, Semnan University of Medical Sciences, Semnan, Iran; 3 Dept. of Oral and Maxillofacial Pathology, Biomaterials Research Center, School of Dentistry, Shiraz University of Medical Sciences, Shiraz, Iran

**Keywords:** Mel-CAM, Mucoepidermoid carcinoma, Oral squamous cell carcinoma, Salivary glands

## Abstract

**Statement of the Problem:**

Mel-CAM (CD_146_, MUC_18_) is a 113-kD heterophilic cell-cell adhesion glycoprotein found in normal and tumoral tissues. The biologic functions and role of the Mel-CAM can be employed as a diagnostic marker in pathology.

**Purpose:**

The aim of this study was assessing the expression of Mel-CAM in common oral carcinomas like salivary gland mucoepidermoid carcinoma (MEC) and oral squamous cell carcinoma (OSCC) to differentiate the OSCC from high-grade MEC.

**Materials and Method:**

This study was performed on 19 specimens of MEC and 17 specimens of OSCC, which were retrieved from the archive of Department of Pathology of Taleghani Hospital, Tehran, Iran. Immunohistochemical staining was performed by using antibody against CD_146_. The data were analyzed using SPSS software through Mann-Whitney, Spearman's correlation coefficient, and Kruskal-Wallis tests.

**Results:**

Mel-CAM was expressed in all MEC samples and 10 OSCC cases. The two groups were significantly different regarding the CD_146_ expression (*p*= 0.035). Furthermore, the CD_146_ expression was found to be significantly correlated with the invasion mode (*p*= 0.002), tumor size (*p*= 0.012), and histological grade (*p*= 0.024) in OSCC group. No significant correlation existed between the expression, intensity, and location with the histological grade of MEC (p&gt; 0.05) nor was any significant correlation detected between the CD_146_ expression and lymph node metastasis in neither group.

**Conclusion:**

Regarding the significant correlation between the CD_146_ expression and the prognostic factors in OSCC, this marker may predict the prognosis in OSCC patients, but not the MEC lesions. It cannot be used for differentiating high-grade MEC and OSCC.

## Introduction

Mucoepidermoid carcinoma (MEC) is one of the most common salivary gland malignancies that mainly affect the parotid. The tumor occurs within the second to the seventh decades of life, and is the most common malignant salivary gland tumor in children [ 1 ]. The biologic behaviors of MEC range from slow-growing mass to destructive rapidly growing mass [ 2 ]. Prognosis of the MEC is usually related to the clinical stage and histological grade [ 3 ]. Despite the advancements in diagnosis and treatment of high-grade MEC over the last two decades, the 5-year survival rate is still less than 50% [ 4 - 5 ]. High-grade MEC can be misdiagnosed for oral squamous cell carcinoma (OSCC), which is the most common malignancy of the oral cavity and accounts for al-most 2% of the cancer burden worldwide. The overall 5-year survival rate has not significantly increased in the last few years despite the advanced treatment modality [ 6 ]. Mel-CAM (CD_146_, MUC_18_) is a 113-kD heterophilic cell-cell adhesion glycoprotein, which belongs to the immunoglobulin supergene family [ 7 ]. It was initially identified as a marker of melanoma progression and metastasis [ 8 ]. This marker is primarily expressed by vascular endothelium and smooth muscle; but has also been detected in subpopulation of activated T lymphocytes, bone marrow, Schwann cells, ductal, and myoepithelial cells of the salivary glands [ 9 ].

Expression of Mel-CAM in tumor tissues is related to the tumor size, progression, metastatic potential, and aggressiveness [ 9 ]. Indeed, the biologic functions and role of the Mel-CAM as a diagnostic marker in pathology are now being recognized. The present study aims to assess the expression of Mel-CAM in salivary gland MEC and OSCC to find its possible correlation with the histological grade, tumor size, lymph node, and metastasis, besides its utility to differentiate the OSCC from high-grade MEC. 

## Materials and Method 

### Sample selection 

The samples of this cross-sectional study were 36 formalin-fixed, paraffin-embedded tissue blocks including 17 cases of OSCC and 19 cases of MEC, which were obtained from the archive of the Department of Pathology of Taleghani Hospital, affiliated to Shahid Beheshti University of Medical Sciences, Tehran, Iran. Anonymity of the patients’ records was strictly respected.

Hematoxylin and eosin (H&amp;E) stained sections were used to confirm the diagnosis. Clinicopathologic information of each case including age, sex, tumor location, and histological grade were collected from the patients' records and reviewing slides. For OSCC samples, mode of invasion was also identified. Cases with incomplete data, insufficient paraffin-embedded tumor material, inappropriate fixation, and incisional biopsy were excluded. 

### Immunohistochemistry (IHC)

Sections of 4-µm thickness were cut from all samples and mounted on silane-coated slides. The sections were deparaffinized with 100% xylene and rehydrated in graded ethanol series. Sections were immersed in Tris-buffered saline (TBS) with a pH of 6.0, and heated in a microwave oven at 750 watts for antigen retrieval. After cooling into room temperature, the sections were incubated with primary antibody (Anti-CD146, monoclonal mouse Anti-Human clone: AA1, Ready to use, ABcam, USA) at 1:2000 for an hour. Having been washed in TBS, the sections were treated with Dako EnVision (Dako, Germany). The DAB chromogen was applied to visualize the antibody expression, and then, counterstained with Mayer’s hematoxylin. Normal parotid salivary gland was used as positive control. 

### Evaluation of IHC

CD_146_ immunoreaction in the tumoral cells was determined in 10 randomly-selected fields by counting all the positive cells in each field according to the median index of positive cells obtained from 10 high-power fields (HPF) and scored as negative (0-5%), weak (6-25%), moderate (26-50%) and strong (51-100%) [ 10 ]. The staining intensity was evaluated as 0=no positive cells, += mild, ++= moderate, +++= strong [ 11 ]. Mode of inv-asion in OSCC samples was assessed on the H&amp;E slides according to Jacobson method (scored I to IV) [ 12 ].

According to WHO classification (2005), the histopathologic grade of OSCC samples was classified into well-, moderate- and poorly-differentiated. The histopathologic grade of the MEC was categorized as low, intermediate and high grade based on Auclair classification [ 10 ]. All slides were evaluated by two pathologists.

### Statistical analysis 

The statistical analysis was carried out on the tabulated data by using SPSS software, version 18.0 (SPSS Inc., Chicago, IL, USA). Mann-Whitney test was used to assess the correlation between CD_146_ expression and the clinicopathologic variables including age, histological grade, nodal metastasis, and mode of invasion. Spearman's correlation coefficient and Kruskal-Wallis test were done to determine the correlation of CD_146_ expression with the tumor size and expression location, respectively. The significance level of all tests was set at 0.05. All the procedures performed in the current study were approved by the Ethics Committee of Shahid Beheshti University of Medical Sciences (#9204) in accordance with the Declaration of Helsinki (1964) and its later amendments. Formal written informed consent was not required with a waiver by the Ethics Committee of Shahid Beheshti University of Medical Sciences.

## Results 

The samples were 23 men and 13 women (Table 1). CD_146_ was expressed in all MEC samples as cytoplasmic and membranous staining. 

**Table 1:** Characteristics of MEC and OSCC patients.

Variables	MEC		OSCC
Sex	Male	10	13
	Female	9	4
	Age(Mean±SD)	45.58±14.31	64.76±9.28
Site of tumor	Alveolar mucosa	6	6
	Parotid	5	
	Sublingual	1	
	Tongue		7
	Hard palate	5	
	Flour of the mouth		2
	Other sites	2	2
Histopathologic grade	High grade	7	3
	Moderate grade	10	3
	Low grade	2	11
Size(Cm) Range (mean)	2.68-3.56 (2.86)		3.5-6.12 (5.08)
Lymph node			
metastasis			
Yes	2 (10.5%)		5 (29.5%)
No	17(89.5%)		12(70.5%)
Mode of invasion*			
I			9
II	-		0
III			4
IV			4

* Mode of invasion was classified only for OSCC

**Figure 1:**
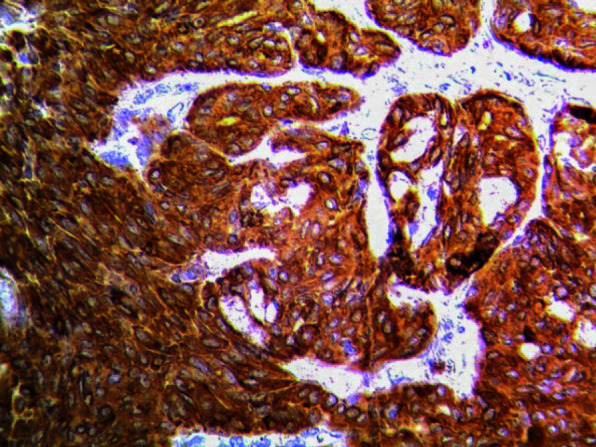
Cytoplasmic and membranous staining of epidermoid cells in mucoepidermoid carcinoma, moderate intensity (CD146 IHC stain, 400 X)

**Figure 2:**
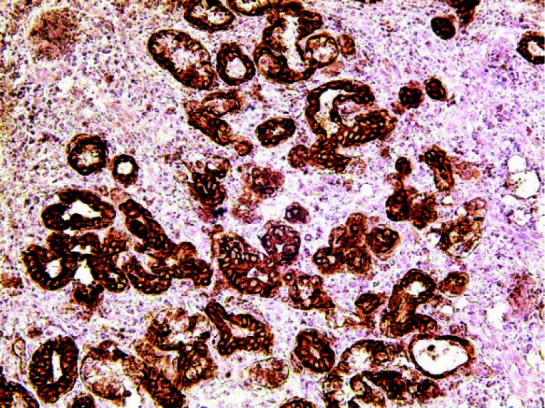
Cytoplasmic and membranous staining of mucous cells in mucoepidermoid carcinoma, strong intensity (CD146 IHC stain, 200 X)

**Figure 3:**
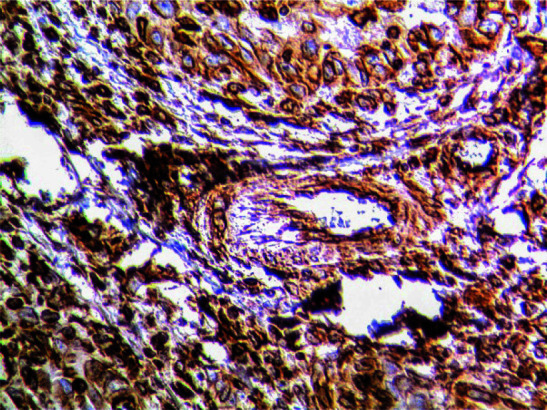
Cytoplasmic and membranous staining of squamous cells in squamous cell carcinoma, moderate intensity (CD146 IHC stain, 400 X)

**Figure 4:**
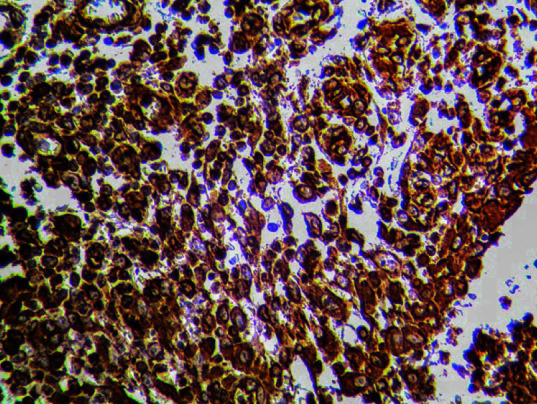
Cytoplasmic and membranous staining of squamous cells in squamous cell carcinoma, strong intensity (CD146 IHC stain, 400 X)

The CD_146_ immunoexpression was strong in 78.9% (n=15), moderate in 5.3% (n=1), and weak in 15.8% (n=3) of cases (Figure 1 and 2). In OSCC samples, 10 cases expressed CD_146_, out of which 47.1% (n=8) was strong and 11.8% (n=2) were weak (Figure 3 and 4). The two groups were significantly different in terms of CD_146_ expression (*p*< 0.035). No significant correlation was detected between the CD_146_ expression, intensity, and location with the histological grade in MEC group (*p*> 0.05). 

However, in the OSCC group, the intensity and expression of CD_146_ was significantly correlated with the histopathological grade of OSCC. Meanwhile, the CD_146_ expression was not significantly correlated with the lymph node metastasis in neither group. Moreover, a significant correlation existed between the CD_146_ expression and the mode of invasion and tumor size in the OSCC cases (*p*< 0.05) (Table 2). 

**Table 2:** Correlation of CD_146_ expression in MEC and OSCC with clinicopathologic findings.

Variables	CD_146_ expression in MEC	CD_146_ expression in OSCC
Metastasis	0.338	0.442
Size	0.649	0.012
Mode of invasion	-	0.002
Histologic grade	0.726	0.024

*p*< 0.05 is significant

## Discussion 

The correlation between the CD_146_ expression and the clinical behavior and lymph node metastasis has been evaluated in some tumors such as melanoma, breast carcinoma, and prostate carcinoma. The present study tried to investigate if the expression of CD_146_ in MEC and OSCC samples is associated with the histological grade, lymph node metastasis, tumor size, and mode of invasion in these tumors. 

In this study, the immunohistochemical expression of CD_146_ was observed in all MEC samples; the staining was strong in 78.9% of samples, which was in accordance with Pires et al.'s findings [ 10 ]. The present study detected no significant correlation between the staining intensity and histological grade of the tumor. It was in agreement with Pires et al.'s study [ 10 ] and in contrast with Zhang’s [ 11 ] and Li’s[ 12 ] findings, who studied the CD_146_ expression in breast carcinoma and malignant cervix tumor. The difference between the studies might be attributed to the type of investigated tumors. 

In high-grade MEC, the staining intensity was moderate in 57.1% and strong in 42.9% of samples. Yet, the correlation between the CD_146_ staining intensity and the histopathologic grade of MEC was not significant. The staining localization was mostly in the cytoplasm of mucous and epidermoid cells. No significant correlation was found between the staining localization and the histopathologic grade in MEC cases. 

CD_146_ was expressed in 58.9% of OSCC cases, which disagreed with what was found by Pires et al. [ 10 ], who reported the CD_146_ expression in none of the OSCC samples in his study. This difference might be due to the different staining method and the smaller sample size in Fabio’s study [ 10 ]. In OSCC samples, strong staining was seen in all tumors with histopathologic grade III, which was statistically significant and in line with Zhang et al.'s study [ 11 ]. 

No significant correlation was detected between the staining intensity and lymph node metastasis (*p*> 0.05). 

Our findings were in contrast with Zhang et al. [ 11 ] and Wu et al. [ 13 ] studies on cervix, ovarian, and prostate tumors. The difference might be due to the different type of tumors and the smaller sample size in current study. Our findings revealed that the CD_146_ expression cannot predict lymph node metastasis in MEC and OSCC but it did in prostate, ovarian tumors, and colorectal cancers [ 14 ]. In addition, Li et al. [ 15 ] noted that the CD_146_ expression was an indicator of poor prognosis in esophageal SCC.

Some studies documented the role of α_7_β_3_ integrin as a ligand of Mel-CAM and accredited that α_7_β_3_ integrin in adults' normal tissues are limited to basolateral membrane cells in ductal epithelium of parotid glands [ 16 ]. Seemingly, the integrin is expressed in normal and benign tumors; but in malignant tumors, it either remains unexpressed or undergoes structural changes, which further lead to dysfunction and lack of connection between the neoplastic cells [ 16 ].

Mesenchymal and epithelial interactions are essential for glandular organ formation in salivary glands. Moreover, the role of CD_146_ in epithelial-mesenchymal transition in breast cancer has been alluded [ 17 ]. Evidence shows that absence or decrease of CD_146_ expression in breast cancer leads to repair connections between the normal cells and waste connections between the tumoral cells, and acts as a suppressor in breast tumor [ 18 ]. The low expression of CD_146_ in normal tissue and benign tumor is used for differential diagnosis of some benign and malignant tumors with similar origin (malignant mesothelioma and reactive one) [ 19 ]. The present study detected a significant relation between the tumor size and CD_146_ expression (*p*= 0.012). OSCC samples of larger than 6.1 cm showed higher expression, which was in accordance with Mills’s in vivo study on melanoma cells [ 20 ]. Other in vivo studies have asserted the role of CD_146_ in aggressive behavior of melanoma cells. Likewise, MMP-2 regulation and inhibition of CD_146_ correlate with less aggressiveness and high apoptotic activity [ 18 ]. The current study detected a significant correlation between the histological grade of OSCC and CD_146_ expression, staining intensity, and localization of staining. These variables had never been evaluated before. Additionally, the mode of invasion was significantly correlated with high expression and intensity of CD_146_ in OSCC, indicating the imperative role of CD_146_ in tumor development and invasion. Regarding the correlation between CD_146_ expression and mode of invasion, the present res ults suggest that CD_146_ expression is a more determining indicator of mode of invasion in OSCC than MEC cases. However, further studies with larger sample sizes may introduce CD_146_ as an advantageous prognostic marker in OSCC. 

## Conclusion

CD_146_ expression is significantly correlated with the mode of invasion, tumor size, and histological grade in oral squamous cell carcinomas. It may help predict the prognosis in patients with OSCC but not MEC. CD_146_ is not a useful marker for differentiating between the high-grade MEC and OSCC. 
